# Experiences of Transgender and Gender Expansive Physicians

**DOI:** 10.1001/jamanetworkopen.2022.19791

**Published:** 2022-06-29

**Authors:** Lauren M. Westafer, Caroline E. Freiermuth, Michelle D. Lall, Sarah J. Muder, Eleanor L. Ragone, Angela F. Jarman

**Affiliations:** 1 Department for Healthcare Delivery and Population Science, University of Massachusetts Chan Medical School–Baystate, Springfield; 2 Department of Emergency Medicine, University of Massachusetts Chan Medical School–Baystate, Springfield; 3 Department of Emergency Medicine, University of Cincinnati College of Medicine, Cincinnati, Ohio; 4 Department of Emergency Medicine, Emory University School of Medicine, Atlanta, Georgia; 5 New York Institute of Technology College of Osteopathic Medicine, Old Westbury, New York; 6 Department of Emergency Medicine, University of California, Davis, Sacramento

## Abstract

**Importance:**

Although LGBTQIA+ (lesbian, gay, bisexual, transgender, queer, and other sexual and gender minority) physicians experience bias in the workplace, there is a paucity of data on the experiences of physicians who identify specifically as transgender and/or gender expansive (TGE; *gender expansive *is an umbrella term encompassing individuals and gender identities that may exist beyond the binary framework [eg, may include nonbinary, genderqueer, and agender individuals]).

**Objectives:**

To explore the professional experiences of TGE physicians, identify barriers to inclusion, and highlight stakeholder-derived strategies that promote an inclusive workplace.

**Design, Setting, and Participants:**

This qualitative study informed by semistructured interviews was conducted among 24 TGE physicians in the US from April 1 to December 31, 2021. The sample of TGE physicians was recruited using convenience and snowball sampling. Interviews were recorded and transcribed. Using thematic analysis, at least 2 members of the research team performed blinded coding of each transcript, in an iterative process.

**Main Outcomes and Measures:**

Data collection and thematic analysis examining themes of physicians’ experiences.

**Results:**

Among 24 physicians (mean [SD] age, 39 [1.4] years) interviewed, 8 (33%) self-identified as transgender women, 7 (29%) as transgender men, 4 (17%) as nonbinary, 3 (13%) as transgender and nonbinary, and 2 (8%) as genderqueer. Prominent themes of the interviews included emotional distress as a result of transphobia, dominance of a rigid binary gender paradigm, and structural and institutional factors that are associated with psychological and physical safety and feelings of isolation as a TGE physician. Clear steps of affirmation were identified that could mitigate the emotional stressors, including signs of safety, active allyship, and mentorship by other TGE physicians.

**Conclusions and Relevance:**

In this qualitative study, TGE physicians reported facing both overt and subtle biases associated with their identity and gender presentation. Participants also noted several interpersonal and structural factors that mitigate the effect of these biases.

## Key Points

**Question:**

What are the professional experiences of US physicians who are transgender and/or gender expansive (TGE)?

**Findings:**

In this qualitative study of 24 physicians who identify as TGE, major themes emerged centering on emotional distress associated with overt and subtle transphobia throughout the spectrum of medical training and practice. A need for structural and interpersonal changes to improve physicians’ understanding of gender diverse individuals and promote workplace inclusivity was identified, with specific examples of mitigation strategies.

**Meaning:**

These findings signal a need for institutions and individuals to engage with strategies to target transphobia and gender biases in the health care setting.

## Teaser

This qualitative study explores the professional experiences of transgender and/or gender expansive physicians, identifies barriers to inclusion, and highlights stakeholder-derived strategies that promote an inclusive workplace.

## Introduction

Lesbian, gay, bisexual, transgender, queer, and other sexual and gender minority (LGBTQIA+) health care workers experience harassment, discrimination, and mistreatment.^1,2,3^ Although approximately 1.2% of matriculating medical students in 2021 identified as transgender and/or gender expansive (TGE; *gender expansive *is an umbrella term encompassing individuals and gender identities that may exist beyond the binary framework [eg, may include nonbinary, genderqueer, and agender individuals]), there is a paucity of understanding of the experiences of TGE physicians, as most studies include few to no TGE individuals (glossary of terms in eTable 1 in the Supplement).^4^ One 2019 survey of 36 TGE medical students and physicians found that 50% experienced barriers attributed to their gender identity when applying for jobs.^5^

Patients may also discriminate against physicians based on their gender expression and/or gender identity, and few institutions have written policies for how this should be addressed. Health care workers have reported increased emotional distress owing to this type of discrimination, which contributes to burnout and can detract from the ability to practice medicine.^1,2,6,7^ It is critical to identify and explore sources and effect of bias toward TGE physicians to mitigate damage and create a more inclusive, healthy environment that promotes retention.

There are limited data characterizing the experience of TGE physicians during training and clinical practice. The objective of this study was to more thoroughly understand the professional experiences of TGE physicians, identify barriers and facilitators of inclusion, and highlight stakeholder-derived strategies that promote an inclusive workplace.

## Methods

### Design

We used exploratory qualitative methods to conduct semistructured interviews with physicians who self-identified as TGE. The research team developed an interview guide, piloted it among 3 TGE individuals, and revised it according to feedback. Interviews began with questions about the participant’s gender identity and gender expression at work. Questions explored the participant’s experiences in training and interactions with colleagues, patients, and administrators. The semistructured interview guide was iteratively revised throughout interviews (eMethods in the Supplement). The study was designed to comply with the Consolidated Criteria for Reporting Qualitative Research (COREQ) reporting guideline for qualitative research. The Baystate Health institutional review board determined the study to be exempt because it was considered to pose minimal risk to the participants. Participants provided verbal consent.

### Study Setting and Population

Physicians who self-identified as TGE were recruited through convenience and snowball sampling from April 1 to December 31, 2021. Study investigators advertised the study through email listservs and on social media (Twitter and Facebook), encouraging any physician in the United States who was TGE to indicate interest in the study via a confidential online registry. At the end of the interview, interviewers asked participants to refer interested contacts who met inclusion criteria to the registry form.

### Data Collection

One interview approximately 35 minutes in duration was conducted with participants and recorded via video conferencing software (Zoom Video Communications) in a setting of the participant’s choosing. Investigators explained the study’s aim of understanding the professional experiences of TGE physicians. Two to 3 members of the research team were present for each interview, based on availability, and took field notes during and after the interviews, which were used to refine the interview guide. Interviews were led by team members with experience with qualitative methods. Demographic information including gender identity, current role (resident physician, fellow, attending physician), practice setting, geographic region, and medical specialty were collected. Although the interview guide structured the interview, participant responses determined the flow and order of the discussion. Interviewers conducted participant checking—ensuring they understood participants’ points and perspectives—in real time, as there was a plan in place to destroy participants’ contact information after the interviews to protect privacy. Participants received a $25 gift card. Interviews were deidentified and transcribed verbatim by a transcription service.

### Research Team and Reflexivity

The 2 interviewers were a cisgender woman emergency physician with extensive experience in qualitative research and a transgender woman emergency medicine resident without existing relationships with participants. Coinvestigators were practicing women emergency physician–researchers and a woman medical student. Seventeen percent of the study team (1 of 6) identified as TGE and 50% (3 of 6) as LGBTQIA+. The research team made it clear at numerous points in the interview that there were no right answers, that answers were confidential, and that participants could skip any questions.

### Statistical Analysis

Demographic data were summarized using descriptive statistics including median values and IQRs, frequencies, and percentages. For privacy, we report medical specialties only if there were 2 or more participants in that specialty. Qualitative data management software was used for coding and analysis of transcripts.^8^ Four team members, including 2 who had extensive experience with qualitative methods, independently reviewed the first transcript and created an initial coding framework using comparison and consensus. Each transcript was coded independently by 2 research team members using this coding framework. The codebook and framework were collaboratively reviewed and refined as new codes emerged or existing codes required clarification. Coding proceeded using a thematic analysis approach and themes were derived from the data.^9^ Codes were iteratively grouped into themes. Thematic saturation, the stage at which codes managed new data without further modification, was reached.^10^ All transcripts were independently recoded by 2 members of the research team once the codebook was finalized. The final codebook can be found in eTable 2 in the Supplement. Steps taken to ensure qualitative rigor can be found in eTable 3 in the Supplement.

## Results

Twenty-four participants (mean [SD] age, 39 [1.4] years) were interviewed: 22 recruited via social media and 2 by snowball sampling. Participants included 8 transgender women (33%), 7 transgender men (29%), 4 nonbinary individuals (17%), 3 transgender and nonbinary individuals (13%), and 2 genderqueer individuals (8%). The Table lists participant demographic information. Two individuals who indicated an interest in the study did not follow up to schedule an interview. Another 2 potential participants did not attend the interview. Thematic saturation was reached after 11 interviews.

**Table.** Participant Demographic Characteristics

Characteristic	Participants, No. (%) (N = 24)
Gender identity	
Transgender man	7 (29)
Transgender woman	8 (33)
Nonbinary	4 (17)
Transgender and nonbinary	3 (13)
Genderqueer	2 (8)
Medical specialty	
Emergency medicine	9 (38)
Family medicine	6 (25)
Surgical specialties	3 (13)
Other nonsurgical specialties	3 (13)
Pediatrics	3 (13)
Provides gender-affirming care^a^	5 (21)
Geography	
South	3 (13)
West	8 (33)
Midwest	5 (21)
Northeast	7 (29)
Mid-Atlantic	1 (4)
Practice setting	
Community	7 (29)
Academic	12 (50)
Hybrid	5 (21)
Career stage	
Medical resident	10 (42)
Early (≤5 y after residency)	7 (29)
Mid (6-14 y after residency)	4 (17)
Late (≥15 y after residency)	3 (13)

^a^ Provides gender-affirming care as a part of their clinical practice in one of the other specialties.

Four major themes emerged, revealing a complex interplay between professional identity, personal identity, and biases in the workplace. The central theme of emotional distress was associated with the dominance of the binary gender paradigm, structural and institutional factors, and affirmations (Figure).

**Figure.**
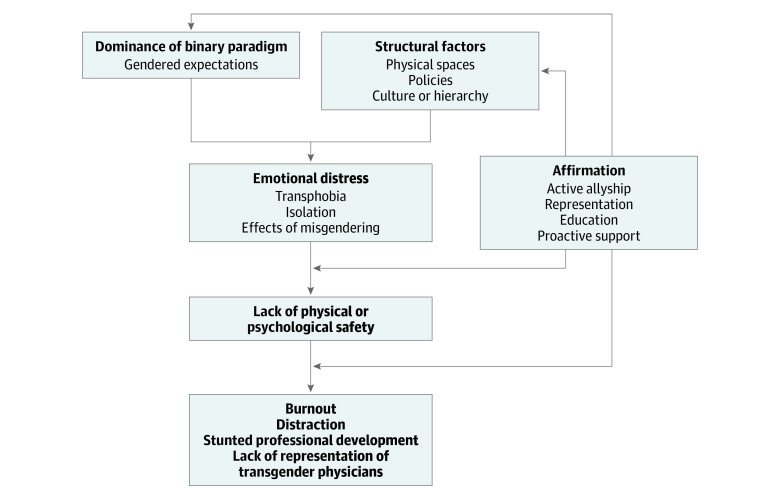
Conceptual Framework All of the arrows coming out of “Affirmation” represent places where affirmational actions can positively disrupt the cycle that feeds into emotional distress.

### Emotional Distress

#### Transphobia

When asked about their professional experiences, participants provided histories in which transphobia caused fear, anxiety, and distress (Box 1). Participants described several sources of transphobia, many beginning with their own difficult experiences as patients and witnessing physicians’ lack of ability or refusal to treat transgender patients. Many participants reported witnessing colleagues be respectful in front of a TGE patient, yet in non–patient-facing arenas engage in misgendering and/or overt bias, typically when colleagues did not know a participant was TGE. In addition, participants highlighted prominent stigma among colleagues, most notably that being TGE was conflated with mental illness. As a result, some participants reported fear of or actual loss of jobs and/or medical privileges.

Box 1. **Illustrative Quotations About Emotional Distress**

**Transphobia**

“I refrained from coming out because I really, really, truly believed on some level that…it simply can’t be done. The messaging that I largely heard, even in medical school, was that like, that concomitant mental illness would make it impossible for a transgender person to ever be sort of successful in a professional setting.” —Participant 13, transgender man
“I had met 2 women who were currently in med school and applied out and had, I mean, just horrible harassment, like admissions departments calling them asking what they thought they were doing applying.”—Participant 24, transgender woman

**Fear of loss of job or license**

“I think, especially for us in health care…it’s a double-edged sword… We don’t get the same privilege of access to mental health care because our records can be subpoenaed, because they can withhold medical licenses and things like that. So, for those reasons, for insurance purposes, I didn’t come out until I was done with everything… I had my life insurance and disability insurance and everything. I was like, ‘you can’t take it from me.’”—Participant 8, transgender woman

**Being out because of obligation to TGE community**

“It’s been hard for me to weigh the balance of wanting to be more open for the sake of helping others, and being sort of a beacon and letting other people know that like yes, this can be done and also not really wanting to deal with that and having fears that my life would be disrupted or people who now very seamlessly are able to navigate my gender identity would you know, suddenly start stumbling where they didn’t before when I was presumed to be cisgender.”—Participant 13, transgender man

**Stress from being “stealth”**

“I have to second-guess everything that comes out of my mouth, because I can’t completely out myself as to why I know something or why I think something. It’s so hard for me to control the fire that explodes in my chest. When someone gets misgendered, it’s like…it’s instant dynamite. And it’s all I can do to keep that controlled enough that I can then put on a show of being a cisgender dude who just happens to feel passionately that you get the patient’s pronouns right and that’s hard to do.”—Participant 16, transgender man

**Timing of transition is complicated in medicine**

“I had 2 rounds of [surgery] within a year because that uncomfortable period in the department with patients not knowing how to address me. I got some frivolous complaints from patients about the ‘weird doctor.’ It was very uncomfortable.”—Participant 8, transgender woman

**Misgendering**

“I think the, you know, feeling of being vaguely misgendered on a regular basis was not nearly as troublesome as having to deal with those conversations on a daily basis.”—Participant 20, nonbinary
“It’s hard at the end of day to have the bandwidth to like go back and bring this up to folks that you know what? It’s actually affecting patient care when you’re traumatizing me dozens of times.”—Participant 17, nonbinary
“I like to use the phrase ‘death by 1000 cuts.’ So, especially the beginning like it was like, oh, it’s not too bad, but then it would just kind of accumulate and wear on you. And then now like now that I’ve been out for a while and somebody misgendered me? It really hurts actually more than I would expect in like, I’m like ‘What is it? Is it my presentation? Is it my voice? Like what’s going on?’… I get kind of distracted.”—Participant 15, transgender woman

**The minority tax—emotional exhaustion**

“Part of me is like, I’m exhausted from the work. And being the person who is from this community, like doing education—it’s emotionally draining. So, I feel like I find myself in both worlds where I want to do the work because I want things to be better. But I also am like, I’m freaking tired of doing this. Can one of you all take this on as your thing, because I’m tired.”—Participant 3, nonbinary

Abbreviation: TGE, transgender and/or gender expansive.

#### Isolation

Also associated with the emotional distress was the feeling of professional isolation that participants described. Most participants noted that they did not know they could be “out” as TGE physicians. Participants noted feeling as if they were the only one, underscoring the importance of TGE representation in medicine and community.

#### Misgendering

Misgendering was common for most participants in the professional setting, who described resultant emotional trauma that, at times, significantly increased their cognitive load. Participants noted pronoun use to be simply a matter of respect and struggled to decide when to address misgendering. They described the emotional toll of constantly policing their identity and the ongoing negotiation of whether correcting an individual who misgendered them would preserve their dignity and emotional well-being without affecting their job. Uniformly, participants reported being less likely to correct patients who misgendered them compared with physicians, administrators, and other hospital staff, citing the desire to focus on the patient. Although being continually misgendered was traumatic, many described correcting pronouns as awkward and rarely resulting in lasting change: “‘This is my name, my pronouns are *they/them*’; I say it at the top of every single case every time and about half of the faculty continued to misgender me all day long.”

#### Challenges Associated With Gender Presentations in the Workplace and/or Transitioning

Although participants reported initiating the process of changing their gender presentation in the workplace during various points in their career, many participants centered their physical and/or professional transition around the timing of training. One-third of participants began their professional transition after the residency or fellowship match to allow for a “fresh start.” However, in each instance, the programs sent out welcome emails with participants’ deadnames (pretransition or pre–coming out name that is often, but not always, the name on their original birth certificate) and/or outdated photos, resulting in unintentionally outing the participants to colleagues. Several participants reported other instances in which they were outed by colleagues or administrators without their consent. In addition, participants noted unique challenges of transitioning while practicing medicine, particularly related to licensure, credentialing, and the health care system’s collective memory of their pretransition gender expression and/or identity. As a result of the sometimes-gradual process of physical transition, participants reported difficulty transitioning while practicing, as some patients lacked trust and confidence in them as physicians based on expected gender norms. Participants noted that the decision to be out as TGE in the professional setting was complicated, emotionally laden, and logistically challenging. They reported a tension between wanting to be “stealth” (ie, “passing,” that is, not known in that setting to be TGE) and being out because of a sense of obligation to be visible for TGE patients and colleagues.

#### Dominance of the Binary Gender Paradigm

Participants overwhelmingly noted that societal expectations and understanding of gender identity and expression as part of a binary paradigm caused emotional distress (Box 2). They described an expectation to conform to a binary model of gender expression by exhibiting traditionally masculine or feminine gender presentations, regardless of their gender identity or preferred modes of expression. They noted that nonbinary gender identities were poorly understood by fellow physicians and patients. Transgender and gender expansive participants reported a penalty associated with nonconformity to the gender binary paradigm, which manifested for some as sacrificing parts of their identity. For example, some nonbinary physicians reported assuming a binary gender identity professionally to avoid conflict and misunderstanding.

Box 2. **Illustrative Quotations About Dominance of the Binary Gender Paradigm, Gendered Expectations, and Structural and Institutional Factors**

**Default is binary and cisgender**

“It’s very much a binary situation, you know, there’s no room for anything in the middle. At this point, it’s safe enough to choose one or the other, but God forbid, you’re in the middle. And, you know, we just don’t really have that many gender-neutral options.”—Participant 7, transmasculine and nonbinary

**Conflation of gender identity with sexuality**

“I did experience some patient hostility around my identity—mostly because I was perceived as a gay man. One time, I was called a faggot. Another time in front of a female intern I was supervising, a patient refused to talk to me because of perceiving me as a gay man and would only speak with the intern. It was very strange to get discriminated against for an identity I did not actually hold.”—Participant 5, transgender man

**Male privilege**

“I think when I presented male, I didn’t get questioned as much. As far as ‘what we were going to do.’ Before a lot of things were taken more at face value. And I have to explain myself a lot more to patients now.”—Participant 8, transgender woman
“I realized pretty early on that I could put on a shirt and a tie and people would take me seriously. And that’s all I had to do. It's the stupidest thing.”—Participant 9, transgender man

**Communication perception**

“When I was a resident, or early on as an attending, if I did the exact same behavior as I do now, I was a ‘bitch.’ But now I’m just an assertive attending who is advocating for his patients and not taking shit from people.”—Participant 4, transgender man

**Structural and institutional factors**

**Institutional culture**

“You come together with these like X number of people for residency and… I never felt like there was a great opportunity to like let people know who I am… I never felt quite comfortable disclosing anything—because the language was never inclusive from the very, very beginning. So, it felt to me to be more of a dangerous space for me to inhabit.”—Participant 2, nonbinary

**Education and competency**

“My primary care said, ‘we don’t know how to take care of your blood pressure, because we don’t know how to take care of people like you.’ It turns out that if you're trans, all anybody has to do is just declare themselves to be an incompetent doctor and they can discriminate against you pretty freely… It’s as common as type 1 diabetes. If you had a type 1 diabetic patient you could read something about it—I can give you the guidelines.”—Participant 11, transgender man

**Physical space**

“I was on a rotation and I had a lot of trouble figuring out which locker room to go into because I was pre-op and I know all these people. So, I had to take 30 minutes out of my time to go find a bathroom that’s more private and then change and then go back in OR scrubs. I’ve had women in the woman's locker room yell at me for being in there because with the mask I pass as a guy but then…at the time, my breasts would have been an issue in the men's locker room to some.”—Participant 7, transmasculine and nonbinary

**Policies**

“I was kind of forced to come out. I was wearing nail polish at work and a patient’s wife had complained that I was wearing nail polish and so I got pulled into my program director’s office with [hospital administrators] and then they were talking about how men couldn’t wear nail polish.”—Participant 1, transgender woman

**Culture**

“Some days just be a little bit like heavier than others where I’m like, wow, did I make the right decision about going into [surgical specialty]? Should I’ve gone into emergency medicine or psychiatry or another field where it just seems like the conversation around identity and sort of professional identity at work are being had…and there are residents that identify across the spectrum. I think that's more of the conversation that I have in my head is—did I make the right choice? Do I belong here?”—Participant 19, nonbinary

**Hierarchy in medicine**

“I found it very difficult to weigh my what I felt to be my duty to speak up against injustice with my desire to remain safe…there’s a lot of power differential… I was very much afraid that not only would there be professional repercussions, but also there might be personal repercussions if I was then sort of suspected as being aligned with the community.”—Participant 13, transgender man

Abbreviation: OR, operating room.

#### Gendered Expectations of Behavior

Participants noted the difficulties of navigating binary gendered expectations. Some participants felt distress associated with navigating the world after changing their gender presentation, largely associated with societal norms in communication styles. They voiced that patients viewed masculine-presenting physicians as authority figures, whereas feminine-presenting physicians were questioned and assumed to not be physicians.

### Structural and Institutional Factors

Many participants described structural and institutional factors that reinforced the binary paradigm and exacerbated emotional distress or actively worked to mitigate 1 or both factors (Box 2).

#### Physical Spaces

Inclusive physical spaces such as all-gender restrooms were identified not only as a basic right (ability to use the restroom) but also as a proxy for an institution’s commitment to inclusion. However, participants noted a dearth of gender-inclusive physical spaces in the health care setting. Those in surgical specialties highlighted the critical importance of these spaces: “explaining to the entire operating room that ‘excuse me, I need 5 extra minutes to go find a bathroom that’s a single stall, that’s not being occupied’ is not something I should have to do a couple of times a day.”

#### Policies

Hospital and professional society policies were identified as reinforcing the binary gender paradigm, at the cost of sometimes outing a TGE physician or causing distress. For example, 1 participant described the requirements for her oral board examination, “It says men—suit, jacket, tie… I’m still listed as male but I hadn’t put a suit on in over a year… I definitely felt upset about it… I just tried to put it in the back of my mind as I was doing the test. I wonder what they expect of nonbinary individuals?”

#### Culture

Participants spoke to the importance of the local culture for promoting inclusion. The binary gender paradigm was often so ingrained in the training program or hospital culture that it required repeated and daily coming out by correcting pronouns on an individual basis, particularly among the nonbinary and gender expansive participants. Some participants wished for a more overtly inclusive culture, which would signal that disclosure was safe. For example, some wished for supported opportunities within their division or program to come out or reinforce their pronouns to avoid repeating this process innumerable times. Participants discussed the hierarchy of medicine, noting that the power differential could be used in an affirming or a traumatic way. For example, participants struggled to correct pronoun misuse among those in positions of power.

### Affirmation

Participants described several interpersonal and institutional factors that mitigated their emotional distress and encouraged inclusion in the workplace (Box 3).

Box 3. **Illustrative Quotations of Affirmational Actions**

**Active allyship**

“We can’t do it alone. We, as transgender humans, nonbinary individuals, historically have been so low on the social ladder—and we're gaining. But at the same time, we can’t do it alone. We can’t necessarily purely advocate for ourselves. And even [if] we do, we need allies. And we need people that, you know, to stand with us and say, ‘Hey, this is not okay.’”—Participant 8, transgender woman

**Proactive pronoun use**

“I think it’s very helpful when it says people use pronouns, display pronouns in meetings and badges to normalize displaying pronouns.”—Participant 18, transgender woman

**Importance of representation in medicine**

“I’ve never seen a health care provider that I could relate to. There was one time I went to, I think, an urgent care, and I saw a PA, that sort of looked like me. And it was like, revolutionary because it was like, I did not think that we made it that far in medicine, like I, it blew my mind.”—Participant 3, transgender and nonbinary
“That’s why I really came out. So that’s been really heartening. And I know that there’s a lot of pain, a lot of providers that are allies that provide really great gender-affirming care. By the same time just having that representation to have a trans or nonbinary patient, point to me and be like, I can trust you, like, immediately, like you, you will see me you will respect me.”—Participant 17, nonbinary

**Education**

“There’s a lot of lack of education. I think, you know, for allies like helping spread the education, and then that also helps us because then we don't have to do all of it.”—Participant 15, transgender woman

**Support from leadership**

“Everybody in the program has been very supportive. My program director actually went with me to my name change hearing as well as all of the program coordinators went with me, our 3 program coordinators went with me, and we went to brunch afterwards and everybody was super supportive.”—Participant 1, transgender woman

**Support from colleagues**

“He was someone that you know, values me as a person and was able to stand up for me in that moment. I have a coresident that has been very helpful in navigating pronouns with my cointerns and will correct people on my behalf, so that I don’t always have to, like, face the burden of that work. So, I think for, for me, like her friendship, and her allyship has been particularly meaningful.”—Participant 19, nonbinary
“I would like to see my colleagues become more inclusive by extending that courtesy to people who they don’t know are trans[gender]… I would like to see a recognition that including and affirming trans[gender] people is not just about being inclusive toward people who you are aware of trans[gender] but also includes a humility that being affirming toward people who you know are trans[gender] doesn’t give you a free pass to assume that everyone else is cis[gender].”—Participant 22, transgender man

Abbreviation: PA, physician assistant.

#### Active Allyship

Active allyship was unanimously identified as a critical way that individuals could support TGE patients and colleagues. Participants gave examples of proactive, affirmative allyship, in which their colleagues interrupted microaggressions such as misgendering and/or transphobic comments in real time. This allyship was particularly powerful when performed by someone in a position of relative power, such as a senior colleague or administrator.

#### Representation

Participants highlighted the need for visible TGE physicians to enhance care of TGE patients and to mitigate the isolation they experienced. Although mentorship might require being out when they otherwise would not share this aspect of their personal life in the professional realm, participants described a desire to help others navigate being TGE as a physician.

#### Education

Many participants described their own difficulty accessing transcompetent health care, even as physicians, and spoke about the need for improved education. In addition, participants believed that education should not be strictly about TGE individuals as patients but also as colleagues. Participants noted a tension, however; although they felt the education to be critical, they were often assumed to be an expert and expected to provide it themselves. They described a “minority tax” and feeling some degree of tokenism but felt “if I don’t do it, nobody else will.”

#### Proactive Support

Participants identified that having proactive support and guidelines sends the message of psychological safety and can mitigate issues associated with transphobia, misgendering, and transitioning. For example, 2 participants noted that their institutions assisted with name changes in documents and electronic health records and aided in communicating the correct name and pronouns to colleagues, which was affirming.

## Discussion

To our knowledge, this study is the first to use semistructured interviews to explore the professional experiences of TGE physicians. Our study confirms and expands previous work demonstrating that health care workers experience significant workplace bias and discrimination based on their LGBTQIA+ identity.^3,11^ The intersection of the major themes is demonstrated in the Figure.

Participants reported a pervasive emotional toll from the consequences of transphobia and a rigid binary gender paradigm, which were often reinforced by structural and institutional factors. The downstream result of this distress and lack of inclusivity is an absence of psychological and/or physical safety that could increase burnout and affect professional satisfaction and success among TGE physicians.^12,13^ Retention of TGE physicians is critical to achieving diversity, equity, and inclusion in medicine, which has been shown to improve patient care, professional mentoring networks, and pipeline programs.^14^ Our study identified strategies for health care systems and training programs, as well as individual physicians, to improve the inclusivity of gender diverse physicians.

Medical schools and institutions should recognize the value in recruiting and retaining TGE students and physicians, including a critical first step of creating psychologically and physically safe environments. Prior literature demonstrates that contact with LGBTQIA+ individuals reduces implicit bias.^15^ This may help combat the transphobia and lack of knowledge witnessed by participants and result in improved patient care.

For TGE individuals to feel safe in medical training and providing care, the physical spaces must be overhauled to include all-gender options for changing spaces and restrooms and ensure these spaces are readily accessible from critical patient care areas (surgical suites, emergency departments, and intensive care units). More important, safety depends on establishing a culture in which transphobia and transexclusion are not tolerated. Health care settings should assess all aspects of recruitment, training, and employment—from first point of contact through the end of employment—and proactively create plans to ensure inclusion. Medical schools, residency programs, hospitals, and health care systems should develop a simplified and standard way for TGE trainees and physicians to go through administrative processes, including licensing and credentialing, using their names and pronouns and eliminate the need to use their deadname. Some institutions have created gender transition tool kits, which could serve as a model.^16^

Transgender and/or gender expansive cultural and clinical competency must be enhanced for substantial cultural shifts to take place. Despite recent efforts to increase LGBTQIA+ health care training, these endeavors are insufficient—the pathologizing of being TGE and conflation of gender diversity with mental illness were commonly reported by our participants.^17,18,19^ In line with prior literature, educational interventions should target transphobia rather than simply provide informational and/or clinical knowledge.^20^ Training programs and institutions should partner with transgender-led organizations that facilitate this education, and the training should span all career stages.^21^

Participants also unanimously emphasized a simple, yet rarely encountered, action with tremendous benefits—speaking up when witnessing misgendering or other transphobic microaggressions or macroaggressions. Participants in our study recounted rare but powerful instances of active allyship. Education of all health care staff on microaggressions and appropriate responses to disrupt these is critical. Facilitator-led, case-based workshops providing guided language for addressing these scenarios may be beneficial. In addition, as suggested by our participants, individuals and institutions can show support and signal safe spaces through the regular use of pronoun pins and by normalizing proper pronoun use.

Although it is important to involve TGE stakeholders in the development and execution of these strategies, it is also imperative to avoid the minority tax.^22^ The responsibility to achieve more inclusive workplaces and TGE physicians cannot and should not be placed solely on TGE stakeholders.

### Limitations

This study has some limitations. Because it is qualitative research, the results should be seen as hypothesis generating. Despite assurances of confidentiality, it is possible that our study may have been subject to social desirability bias. In addition, most of our study team were emergency medicine physicians, and all were women. Although we attempted to ensure rigor and routinely discussed reflexivity at meetings, it is possible that these characteristics introduced bias into our study.

## Conclusions

In this qualitative study, TGE physicians reported stigma associated with their gender identity and presentation, leading to emotional distress in the professional setting. The findings suggest that institutions and physicians must take steps to promote inclusivity at both the organizational and interpersonal levels.

## Notes

Supplement. **eTable 1.** Glossary of Terms **eMethods.** Interview Guide **eTable 2.** Codebook **eTable 3.** Qualitative Rigor
eReferences.
