# Supplementary material for: Experiences of Transgender and Gender Expansive Physicians
Source: JAMA Netw Open. 2022 Jun 29;5(6):e2219791. doi: 10.1001/jamanetworkopen.2022.19791 (PMC9244607; doi:10.1001/jamanetworkopen.2022.19791)
Supplement: Supplement. — eTable 1. Glossary of Terms eMethods. Interview Guide eTable 2. Codebook eTable 3. Qualitative Rigor eReferences. [file jamanetwopen-e2219791-s001.pdf]

Supplementary Online Content

Westafer LM, Freiermuth CE, Lall MD, Muder SJ, Ragone EL, Jarman AF. Experiences of transgender and gender expansive physicians. *JAMA Netw Open.* 2022;5(6):e2219791. doi:10.1001/jamanetworkopen.2022.19791

eTable 1. Glossary of Terms
eMethods. Interview Guide
eTable 2. Codebook
eTable 3. Qualitative Rigor
eReferences.

This supplementary material has been provided by the authors to give readers additional information about their work.

© 2022 Westafer LM et al. *JAMA Network Open.*

eTable 1. Glossary of Terms

Assigned gender at birth (AGAB)	A recorded gender (gender represented by various legal or other institutions/systems) generally indicated by the gender on the birth certificate.[1]
Gender	Gender comprises the social, environmental, cultural, and behavioral factors and choices that influence a person's self-identity
Gender identity	An individual's sense of belonging to a gender category as male, female, a blend of both, neither, or another gender and is comprised of both biological and sociological elements[1]
Gender expression	The external manifestation of categorized gender norms based on sociocultural context (appearance, dress, and behavior)[1,2]
Cisgender	A term used to describe a person whose gender identity aligns with those typically associated with the AGAB
Transgender	An umbrella for people whose gender identity and/or expression is different than the cultural expectations based on their AGAB and may include gender nonbinary, gender nonconforming, or genderqueer identities.[3] Note: being transgender does not imply a specific sexual orientation.
Transgender woman	Typically used to describe a woman who was assigned male at birth
Transgender man	Typically used to describe a man who was assigned female at birth
Non-binary	This is an umbrella term used for gender identities outside of the gender binary (man/woman) but does not mean the same as "intersex". Individuals who identify as non-binary may identify as an intermediate or separate gender, identify with more than one gender or have fluctuating gender identity (genderfluid), or identify as agender. Some, but not all, individuals who identify as non-binary classify themselves as transgender.[4]
Genderqueer	A person who blurs preconceived binary gender boundaries and may identify as a combination of gender, neither, or both.[3]
Gender expansive	An umbrella term encompassing individuals and gender identities that may exist beyond the binary framework (e.g. may include non-binary, genderqueer, agender)[5]
Coming out	The process of disclosure of gender identity to oneself and/or others. There are degrees of being out (to self/social circles/professionally), and coming out is a personal, lifelong process.[3]
Transition	This term refers to the process a transgender individual goes through to affirm their gender identity and may occur in social, legal, and/or medical realms. The process of transitioning may include some of the following: changing names / pronouns / identification documents, as well as hormones and/or surgeries. Not everyone who identifies as transgender will undergo transition, or chose to transition in the same ways (e.g. hormone therapy, surgery, etc)[3]
Passing	A term used to describe when someone is perceived as their correct gender and gender-typical / cisgender.[3]
Stealth	A term used to describe transgender or gender-expansive individuals who maintain their privacy and do not disclose (similar to passing).[3]
Gender affirming care	Health care that attends to transgender individual's physical, mental, and social health needs and well-being while respectfully affirming their gender identity
Misgendering	To refer to someone using a word, especially a pronoun or form of address, which does not correctly reflect their gender. This may be unintentional and without ill intent or can be a maliciously employed expression of bias. Regardless of intent, misgendering has a harmful impact.[3]
Deadname	A term used by some transgender individuals to refer to their pre-transition or pre-coming out name which is often, but not always, the name which appears on their original birth certificate (i.e.birth name or "assigned name"). Deadnaming is the practice of referring to an individual by their deadname and is considered a form of abuse.[1]

© 2022 Westafer LM et al. *JAMA Network Open.*

eMethods. Interview Guide

Interview Guide

Thank you so much for agreeing to participate in this interview. We are conducting a study of the professional experiences of emergency physicians who identify as transgender a-nd/or non-binary. We are going to ask questions about biases, gender biases, and transphobia in the workplace. Please skip any questions you don't want to answer. You can also ask to stop the interview at any time. As mentioned previously, all information will be de-identified and in no way linked to you.

To help us understand the intersection of your experiences with gender and your professional career, it would be helpful to hear a bit about your gender identity and how you currently present at work.

And has this changed over time? / Have you always presented this way at work?

Tell me a bit about how

Do colleagues identify you or do you identify yourself as transgender and/or non-binary in the workplace?

How do you think this is perceived in your current job?

How does this affect your interactions with colleagues?
Students?
Patients?

If participant began presenting in their identified gender after the beginning of medical school, ask the following questions….

- Have you experienced any changes in patients' confidence in you as an emergency physician before and after transitioning/presenting as [ ]?
- Do you feel like your own confidence as an emergency physician has changed before and after transitioning/presenting as [ ]?
- Have you experienced any changes in how patients perceive your 'likability'?
- Have you experienced any changes in how colleagues perceive your 'likability'?
- Do you feel there has been a change in your professional evaluations since transitioning/presenting as [ ]? (from supervisors, trainees, nurses, patients)

Tell me about any biases you've experienced in medical school, in training, or in your career as a physician.

- Tell me about any experiences with violence, aggression, or microaggressions

Do patients frequently misgender you?

- Tell me about how you react to that...
- Does it come more from males/females/or anyone?

Do patients correctly gender you on a regular basis?

- Tell me about how you react to that...

What about colleagues, do they frequently misgender you?

- How do you react when that happens?

What about colleagues, do they frequently correctly gender you?

- How do you react to that?

How has representation from transgender and/or non-binary emergency physicia-ns played a role in your career and gender identity at work?

- Do you feel this is something that needs significant changes?
- Do you perceive yourself taking on such a role throughout your career?

Have you had direct or indirect pressure to avoid pursuing leadership positions (whether locally, nationally, etc.) as a result of your gender identity?

How have witnessed experiences towards transgender or non-binary individuals from other providers affected your perception of emergency medicine?

- Has it changed the way you provide care?
- Has it changed the way you educate / train / lead?

© 2022 Westafer LM et al. *JAMA Network Open.*

eTable 2. Codebook

**Domains (Bold) / Themes / Subthemes (Indented)**
**Affirmation**
Active allyship
Normalize talking about pronouns/gender identity
Opt-in pronoun disclosure (badges/posters)
Pronoun pins invite conversation
Speak up
being 'accepted' but not 'understood'
connecting with other marginalized groups
Equality departments
Intake structure asks pronouns, sex assigned at birth
Obligation to TGNB community
Online community
opt-out therapy for clinicians
Pro-active guidance
Include TGNB in planning process
Prepare to support potential for trans residents
Professional education on TGNB
Representation is important
Colleagues perform differently in front of TGNB colleagues
Important to disclose gender identity at times to align with patients
Insufficient education/Lack of representation/inclusion in MedEd
Lack of representation/space in professional societies
Need for mentors in specialty/medicine
Role of LGB / queer mentors/community in specialty/medicine
Seeing someone 'like them'
trans/NB patients seek trans/NB clinicians
Support from colleagues
Superficial support undermines progress
Support even when trans individual isn't visible
Support from administrators/attendings
Ease of name change, etc
Support from fellow residents/students
**Fear/Anxiety/Stress**
Fear in seeking healthcare
How to Disclose
Lack of capable healthcare clinicians
Prior trauma
Privacy
Fear of loss
Job loss

© 2022 Westafer LM et al. *JAMA Network Open.*

Difficult to prove discrimination
More difficult in training
On Being 'Out"
Don't come out as nonbinary because complicated
Forced to be 'out'
Have to come out due to name/pronoun change
Outed by Colleague
To ensure fit/lack of discrimination
Important to be oneself
No clear way to 'come out' professionally
out after match
'Out" out of obligation to TGNB community
To support patients
Prefer to be stealth
selectively 'out' based on others around
not out with conservative patients
Personal taxation "the minority tax"
Depersonalization
emotional exhaustion
if I don't do it - who will?
Looking for helpful allys to take on work
Isolation
Stress from being stealth
Transphobia
Difficulty finding healthcare
Gay/lesbian privilege
Internalized Transphobia
microaggressions
Overt transphobia
Bias against trans women
Intentional (to be harmful)
Particularly if don't fit typical model
religiosity perpetuating transphobia
Seen as deviant or odd /psych
Present in training
Safety
Seen as 'difficult to get along with'
trans exclusive feminism
Transphobia leading to burnout
Witnessed against patients
**Gendered expectations**
assigned roles that don't align with interests

© 2022 Westafer LM et al. *JAMA Network Open.*

assumption of gender experiences
Binary gender
Binary easier to understand
Default is binary and cisgender
Pressure to conform to gender norms
Communication
change in communication and perception
Male voice "heard" more
Expected gender norms
Categorized based on what people can relate to (gay/lesbian)
Have to be careful with kids as male
Male as machismo
More empathy as female
lack of exposure to gender diversity
Male privilege'
Assumption that female=nurse, male = leader/doctor
Bar lower for men
Sexual harassment as female
Tension between affirmation and frustration with gender affirmation & sexism
Woman treated differently by nurses
Misgendering
Addressing misgendering
Confrontation
Dilemma of when to correct
Quick acknowledgement makes people feel safe
Affects work
Asking pronouns isn't enough
Avoid correction to avoid getting into a conversation
Can open up to liability
Culture can help reduce misgendering
Euphoria/surprise when correctly gendered
Not a big deal when done by patients
Pronoun use culturally ingrained
Repeat offenders
Passing' privilege
**Transitioning**
Changes in evaluations/criticism of performance
Emotional Changes
Hard
Difficult to find care
Hard to go through in front of people
Improved performance once true to oneself

© 2022 Westafer LM et al. *JAMA Network Open.*

Legal issues
nurse distrust of care
Timing of transition
Fear that transition will affect evaluation/job
Post-match/ acceptance
Power dynamics
Time transition for "fresh start"
Deadnaming/misgendering starts with welcoming
Transition at same place can leave a shadow
Transition as physician is complicated
Transition is gradual
Peri-transition awkwardness
Patient distrust
**Institutional/Structural Issues**
Culture
Specialty choice
Presence of queer/non-trans colleagues
Provide gender affirming care (demonstrates support)
How TGNB patients are treated (e.g. rooming issues)
Hierarchy can perpetuate biases
Job opportunities
Seek inclusive environment
Disconnect between enthusiasm for candidate and action taken
Lip service to inclusion
Provide gender affirming care (demonstrates support)
Side conversations/comments can be hurtful
Leadership of TGNB clinicians
Become a safety net for patients
Take on educational leadership
Tokenism
EHR perpetuating dead name/misgendering
Difficult to change
Lack of understanding of when sex assigned at birth is important
No momentum to change
Gender behavior Institutionalized by policy/norms
dress/code professionalism standards
Not built for non-binary
Physical space issues (locker rooms/bathrooms)
Presence of gender-neutral restrooms (affirming)
Role of Geography (rural/South less inclusive)

© 2022 Westafer LM et al. *JAMA Network Open.*

eTable 3. Qualitative Rigor

	Purpose	Strategies applied in our study to achieve rigor
**Credibility**	To establish confidence in the results	Interview protocol (guide and process) tested in 3 pilot interviews
		Trained primary interviewer and investigators
		Member checking – Gives participants opportunity to correct errors and challenge what are perceived as wrong interpretations
**Dependability**	To ensure the findings are repeatable within the same cohort of participants, coders, and context	Rich description of the study methods: We created a detailed draft of the study protocol
**Confirmability**	To extend confidence that the results were derived from the data and would be confirmed by other researchers	Reflexivity: Although our study team was comprised of women emergency physicians/trainees and one student, we included investigators who are members of the LGBTQIA+ community and not, and members across the spectrum of training/career. We also reported research positions and demographics in the manuscript.
		Triangulation: We triangulated sources and examined the consistency of individuals with different backgrounds, gender identities, and specialties
**Transferability**	To extend the generalizability or transferability of results to other contexts/settings	Sampling to form a nominated sample: We used a combination of sampling techniques (recruited via social media, list servs, and by word of mouth).
		Data Saturation: We identified when we reached theoretical data saturation and extended interviews to reach demographic saturation targets (gender identity / region of United States)
		Thick description: we used probes in the semi-structured interviews to enhance description

© 2022 Westafer LM et al. *JAMA Network Open.*

eReferences.

1. Kronk CA, Everhart AR, Ashley F, et al. Transgender data collection in the electronic health record: Current concepts and issues. *J Am Med Inform Assoc*. 2022;29(2):271-284.

2. Stahel D. Glossary of terms. Human Rights Campaign. Accessed April 29, 2022. https://www.hrc.org/resources/glossary-of-terms

3. LGBTI-SafeZone terminology. Accessed April 28, 2022. https://www.edi.nih.gov/people/sep/lgbti/safezone/terminology

4. Understanding non-binary people: How to be respectful and supportive. National Center for Transgender Equality. Published July 9, 2016. Accessed April 27, 2022. https://transequality.org/issues/resources/understanding-non-binary-people-how-to-be-respectful-and-supportive

5. What is gender diversity? A Gender Agenda. Published July 19, 2019. Accessed April 28, 2022. https://genderrights.org.au/information-hub/what-is-gender-diversity/

© 2022 Westafer LM et al. *JAMA Network Open.*
